# Does Diabetes Matter? The Efficacy of PRP on the Quality of Life in Stress Urinary Incontinence

**DOI:** 10.3390/bioengineering12111179

**Published:** 2025-10-29

**Authors:** Andreea Borislavschi, Răzvan-Cosmin Petca, Aida Petca

**Affiliations:** 1 Department of Obstetrics and Gynecology, “Carol Davila” University of Medicine and Pharmacy, 8 Eroii Sanitari Blvd., 050474 Bucharest, Romania; andreea.borislavschi@drd.umfcd.ro (A.B.); aida.petca@umfcd.ro (A.P.); 2 Department of Obstetrics and Gynecology, Elias University Emergency Hospital, 17 Mărăști Blvd., 050474 Bucharest, Romania; 3 Department of Urology, “Carol Davila” University of Medicine and Pharmacy, 8 Eroii Sanitari Blvd., 050474 Bucharest, Romania; 4 Department of Urology, “Prof. Dr. Th. Burghele” Clinical Hospital, 20 Panduri Str., 050659 Bucharest, Romania

**Keywords:** stress urinary incontinence, diabetes, PRP treatment, regenerative treatment in gynecology, SUI treatment

## Abstract

**Background:** Stress urinary incontinence (SUI) is quite common in women, impairing the quality of life. Diabetes mellitus may exacerbate pelvic floor dysfunction and alter treatment response. Platelet-rich plasma (PRP) is a regenerative option to treat SUI; however, data in women with diabetes are limited. This prospective, non-randomized comparative cohort study evaluated PRP outcomes in diabetic versus non-diabetic women with SUI. **Methods:** Women with SUI (n = 102; non-diabetic n = 80, diabetic n = 22) received up to three PRP injections at 4–6-month intervals. They were evaluated using the Stamey scale before and after treatment, via the King’s Health Questionnaire (KHQ), assessed at baseline and after each session. Within-group change was tested using Friedman’s test with Dunn–Bonferroni post hoc comparisons; between-group differences used Mann–Whitney U tests. **Results:** PRP was associated with significant improvements across KHQ domains, with the largest gains by the second injection and smaller increments thereafter. Non-diabetic participants showed earlier, more uniform improvement and additional gains from post-PRP1 to post-PRP2/3. Diabetic participants improved later, with fewer between-session differences. Regarding the Stamey scores, our study concluded that diabetics and non-diabetics improve with PRP treatment, whereas the diabetics treated with insulin have not reached statistical significance in improving SUI. **Conclusions:** PRP was associated with meaningful, multi-domain improvement in women with SUI, with the most benefit by the second injection and continued reduction in symptom burden thereafter. Although patients with diabetes improved, they had higher total KHQ scores at baseline and later visits, supporting tailored counseling and potential protocol optimization for this subgroup.

## 1. Introduction

Stress urinary incontinence (SUI) remains challenging to treat in women, especially among those with diabetes mellitus. Traditional approaches for SUI include conservative therapies, medication-based management, and surgical procedures. However, emerging therapeutic avenues such as Platelet-Rich Plasma (PRP) therapy have invoked interest owing to their regenerative potential and minimal invasiveness [1].

Urinary incontinence is defined as the involuntary loss of urine, and it is classified as urgency urinary incontinence (UUI), SUI (during activities that increase abdominal pressure, such as coughing, sneezing, or exercising), or mixed urinary incontinence (MUI) [2]. Recent advances in the treatment of SUI include mobile health applications for pelvic floor muscle training, medication (selective serotonin reuptake inhibitor), functional magnetic stimulation (to improve contractility of pelvic floor muscles), and interventions to stimulate tissue elasticity and regeneration (PRP, autologous stem cell transplantation, laser therapy, and radiofrequency treatment) [2].

The symptoms of SUI place a considerable burden on the quality of life (QoL) with impactful consequences such as social embarrassment, restrictions on daily activities, and emotional burden, including depression and anxiety [3]. Statistics highlight a prevalence of 37.5% for SUI in women; a study concluded after evaluating 5006 adult U.S. women [4]. SUI is often underreported due to embarrassment, stigma, and the belief that it is a normal consequence of aging or childbirth. This reluctance to seek medical care might result in underdiagnosis; therefore, the true prevalence may be higher than current estimates [3].

The literature indicates that diabetes in women is frequently linked to impaired pelvic floor function, primarily due to neuropathic and microvascular alterations. Reported associations include diabetes with urgency urinary incontinence (UUI), mixed urinary incontinence (MUI), and stress urinary incontinence (SUI), with poor glycemic control identified as a contributing risk factor for SUI [5,6,7,8].

Several clinical studies have evaluated the effectiveness of PRP treatment for SUI, demonstrating improvements in symptom scores and QoL measures [1,2,3,9,10]. However, the existing data on SUI treatment rarely isolates diabetic women, mainly for methodological and practical reasons, and to date, no published trials or case series have specifically investigated the efficacy of PRP treatment in this category of patients. Considering the known negative impact of diabetes on the healing processes, it remains unclear whether PRP offers comparable therapeutic benefits in this subgroup, and the adoption of minimally invasive regenerative treatments for these patients still remains an unmet need.

The present study addresses this gap of knowledge by prospectively comparing the outcomes of PRP therapy for SUI in diabetic and non-diabetic women, with the primary objective of determining whether diabetes influences treatment effectiveness, especially in the QoL, as diabetics already belong to a vulnerable minority. Secondary objectives included evaluating overall symptom improvement and safety in both groups.

Given the current literature indicating that PRP therapy for SUI improves QoL and reduces symptom severity, the aim of this prospective, non-randomized, comparative cohort study was to determine whether treatment outcomes differ between those with and without diabetes mellitus. By comparing changes in quality of life, as measured by the King’s Health Questionnaire (KHQ), and also using the Stamey scale (Stamey, 1980) [11], between these two cohorts, the study sought to clarify the potential influence of diabetes-related pathophysiological factors on PRP efficacy [12,13].

## 2. Materials and Methods

### 2.1. Study Design and Setting

This prospective, non-randomized, comparative cohort study was conducted at Elias Emergency University Hospital, Bucharest, Romania, between April 2022 and May 2025. The study included women diagnosed with SUI treated with PRP according to a standardized protocol. Participants were stratified into two groups based on the presence or absence of diabetes mellitus. The study was designed to compare treatment outcomes between these groups and to evaluate overall symptom improvement and adverse events. Ethical approval was obtained from the institutional ethics committee (Approval No. 2449/23.03.2022), and all participants provided written informed consent before enrollment.

### 2.2. Participants

**Figure 1:**
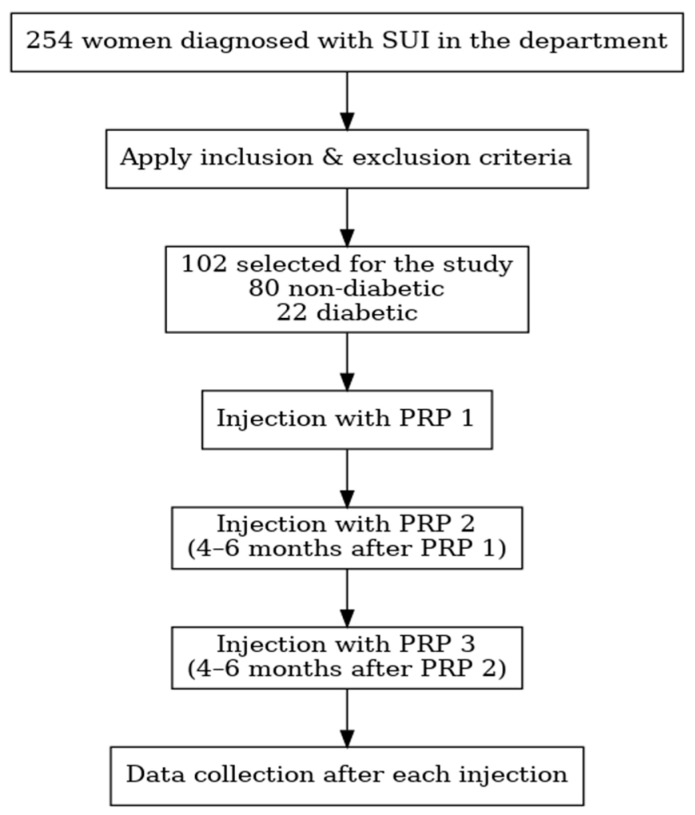
Steps of the study. Flowchart illustrating patient selection and study design. A total of 254 women diagnosed with stress urinary incontinence (SUI) were screened. After applying inclusion and exclusion criteria, 102 participants (80 non-diabetic and 22 diabetic) were enrolled. Each participant received three platelet-rich plasma (PRP) injections administered 4–6 months apart, with data collection performed after each injection. PRP: platelet-rich plasma; rpm: rotations per minute; SUI: stress urinary incontinence.

A total of 254 women were diagnosed with SUI in the department of Obstetrics and Gynecology at Elias Emergency University Hospital, Bucharest, Romania. Eligibility was determined through patient-reported symptoms corroborated by clinical and gynecological examination (including the Valsalva stress test as the standard initial clinical assessment) (Figure 1).

After applying the inclusion and exclusion criteria (Table 1), a total of 102 women were included in the study and underwent PRP therapy according to a standardized protocol. Of these, 22 participants (21.6%) had a confirmed diagnosis of diabetes mellitus type 2, while 80 (78.4%) were non-diabetic. All patients received three periurethral PRP injections at 4–6-month intervals, prepared and administered using a uniform technique. Baseline demographic and clinical data were collected, and quality of life was assessed using the King’s Health Questionnaire (KHQ) at baseline, before each injection, and after the final injection [13]. The Stamey scale was also used pre- and post-treatment to quantify the urine loss [11]. The primary objective was to compare treatment outcomes between diabetic and non-diabetic women, with secondary analyses evaluating overall symptom improvement and adverse events (Table 2). Permission to use the King’s Health Questionnaire (KHQ) was obtained from Mapi Research Trust through ePROVIDE™ and administered in accordance with the terms of the User License Agreement (No. 120730).

The study design prioritized patient-reported outcomes to assess the therapeutic impact of PRP on quality of life and symptom perception, rather than focusing on the quantification of urine loss. Therefore, objective tests such as the pad test were not included. The Stamey scale evaluated the severity of SUI before and after treatment. The KHQ was selected as a validated and comprehensive instrument for evaluating treatment response, with Questions 9 and 10 serving as semi-objective measures reflecting symptom frequency and severity.

All participants underwent standard laboratory testing prior to each PRP injection, including a complete blood count, liver transaminases, and fasting blood glucose. In all cases, fasting glucose levels were within the normal range and platelet counts were consistently normal. At study entry, information obtained from the patients’ medical records and clinical history confirmed that diabetes was well controlled, with glycated hemoglobin (HbA1c) values not exceeding 6.5%. Overall, all laboratory parameters remained within normal limits throughout the study period, indicating metabolic stability.

### 2.3. Intervention Protocol

**Figure 2:**
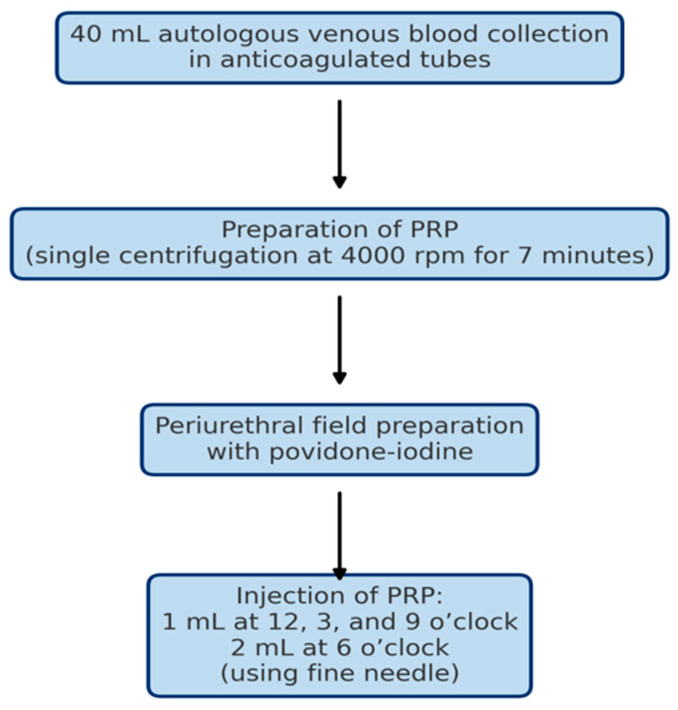
PRP procedure and preparation. Schematic representation of the platelet-rich plasma (PRP) preparation and injection procedure. A total of 40 mL of autologous venous blood was collected in anticoagulated tubes and centrifuged at 4000 rpm for 7 min. After periurethral field preparation with povidone-iodine, PRP was injected periurethrally using a fine needle: 1 mL at the 12, 3, and 9 o’clock positions, and 2 mL at the 6 o’clock position. PRP: Platelet-rich Plasma; rpm: rotations per minute; mL: milliliters.

PRP was prepared from 40 mL of autologous peripheral venous blood collected into sterile, anticoagulated tubes. The samples were processed using a single centrifugation step at 4000 rotations per minute for 7 min (all patients received the same protocol of PRP preparation to ensure homogeneity). The PRP was prepared without the use of a commercial kit. Following centrifugation, the upper plasma layer, together with the buffy coat, was aspirated from the anticoagulated tube using a sterile syringe, ensuring minimal disturbance of the underlying red blood cell layer, resulting in approximately 5 mL of PRP (Figure 2).

Under aseptic conditions, following periurethral field preparation with povidone-iodine, PRP was administered according to a standardized injection protocol: 1 mL at the 12, 3, and 9 o’clock positions, and 2 mL at the 6 o’clock position. A fine-gauge needle was used to minimize patient discomfort.

Each patient received a total of three treatment sessions, scheduled at 4–6-month intervals. The chosen interval allowed for tissue response and potential regenerative effects between sessions. Patients underwent periurethral infiltration with 2% lidocaine for local anesthesia. Patients were observed briefly post-procedure before being discharged the same day. Post-procedure instructions included avoiding sexual activity, tampon use, and vigorous physical exercise for 48 h. Patients were advised not to use NSAIDs for at least 2 weeks before and after the procedure and to hydrate properly (30–50 mL/kg body).

### 2.4. Outcome Measures

The primary outcome measure was the change in QoL from baseline to the first, second, and third PRP injections, assessed using the King’s Health Questionnaire (KHQ), and also using the Stamey scale pre- and post-treatment [13]. The KHQ is a validated, disease-specific instrument widely used for evaluating QoL in patients with urinary incontinence. It covers multiple domains, including general health perception, incontinence impact, role limitations, physical limitations, social limitations, personal relationships, emotions, sleep/energy, and symptom severity. The KHQ is available for non-commercial research without licensing fees and has been validated in multiple languages, ensuring applicability across diverse populations. The Stamey scale (Stamey, 1980) [11] is a widely used clinical grading system designed to evaluate the severity of SUI. It provides a simple, standardized measure of symptom intensity based on the circumstances under which urine leakage occurs. The scale is typically graded from 0 to 3, where grade 0 indicates continence with no leakage, grade 1 represents leakage only during vigorous activity such as coughing, sneezing, or exercising, grade 2 denotes leakage with minimal physical exertion or walking, and grade 3 reflects continuous or spontaneous leakage without effort. The Stamey scale is particularly valuable for assessing baseline symptom severity and for tracking treatment response over time, as it correlates well with patient-reported outcomes and quality-of-life measures in women with SUI.

Secondary outcomes included patient-reported symptom improvement and the occurrence of adverse events, recorded at each treatment visit.

### 2.5. Statistical Analysis

We summarized each KHQ domain and the total score pre-PRP and post-PRP1/2/3 using statistics (mean ± SD, median [IQR], and minimum–maximum). We also evaluated the Stamey scale pre- and post-treatment regarding the entire cohort and subgroups. Given the ordinal scaling and non-normal distributions, within-group changes across visits were tested with the Friedman test; when significant, we performed Dunn post hoc comparisons with Bonferroni adjustment and reported the adjusted *p*-values. Between-group differences (diabetic vs. non-diabetic) at each time point were assessed with the Mann–Whitney U test (two-sided α = 0.05); exact *p*-values and effect sizes (r) are provided where available. Analyses used complete cases without imputation and were conducted in DATAtab (datatab.net, version 2.3.2.).

## 3. Results

**Figure 3:**
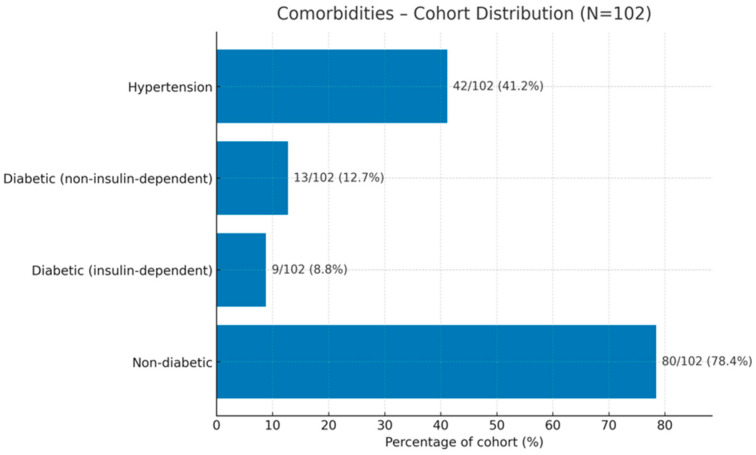
Comorbidities in the entire cohort and subgroups. Distribution of major comorbidities among participants. The majority of the cohort were non-diabetic (76.4%), followed by arterial hypertensive patients (41.2%), non-insulin-dependent diabetics (12.7%), and insulin-dependent diabetics (9.8%). Values are expressed as the number and percentage of patients within the total study population. N: number.

The cohort comprised 102 women (mean age 58.6 ± 10.8 years; range 38–82) (Table 3) with a mean parity of 1.89 ± 0.94 births (range 0–4). Most were non-diabetic (80/102, 78.4%), with 22/102 (21.6%) diabetic; among diabetics, 9/22 (40.9%) were insulin-dependent and 13/22 (59.1%) were not. In total, 37 participants were current smokers (36.3%) and 42 had hypertension (41.2%). The majority were parous (97/102, 95.1%); among parous women, 90/97 (92.8%) reported vaginal delivery only, 4/97 (4.1%) Caesarean only, and 3/97 (3.1%) both modes (overall distribution including nulliparas: vaginal 88.2%, Caesarean 3.9%, combined 2.9%, nulliparous 4.9%) (Figure 3).

### 3.1. KHQ Scores and Domains

(a) Question 1

Question 1 of the King’s Health Questionnaire (KHQ) assesses participants’ perception of their general health.

In the overall study population, the score progressively decreased from a pre-PRP mean of 45.59 points to post-PRP, with a gradual decline from 31.37 at post-PRP1, to 23.04 at post-PRP2, and 18.14 at post-PRP3 (*p* < 0.001). Among the non-diabetic subgroup, the mean score decreased progressively from 45.00 (pre-PRP) to 30.31 (post-PRP1), 20.31 (post-PRP2), and 15.97 (post-PRP3) (*p* < 0.0001). All pairwise comparisons between pre-PRP and post-PRP time points showed statistically significant differences (*p* < 0.001, *p* < 0.001, *p* < 0.001, respectively), as well as between post-PRP1 scores and post-PRP2 and -3 (*p* = 0.003, *p* < 0.001, respectively). However, no statistically significant differences were observed between the post-PRP2 and post-PRP3 scores (*p* = 0.794). In the diabetic subgroup, the scores showed a statistically significant overall change across the four time points (*p* = 0.003). No significant differences were observed between the pre-PRP and post-PRP1 scores (*p* = 0.123); however, significant improvements were recorded after the second and third PRP sessions (*p* = 0.034 and *p* = 0.001, respectively). No statistically significant changes in the score were noted between post-PRP1, post-PRP2, and post-PRP3 scores (Supplementary Table S1).

Inter-group analysis revealed no significant differences between the diabetic and non-diabetic groups for pre-PRP and post-PRP1 mean scores (*p* = 0.401, *p* = 0.297, respectively). Yet, post-PRP2 and post-PRP3, non-diabetic patients reported significantly lower mean scores compared to their diabetic counterparts (*p* = 0.018, *p* = 0.021, respectively).

(b) Question 2

Question 2 of the King’s Health Questionnaire (KHQ) evaluates the extent to which urinary incontinence interferes with daily life.

In the overall study population, the responder’s scores showed a significant decline from a pre-PRP mean of 65.69 to 49.02 post-PRP1, 35.95 post-PRP2, and 30.72 post-PRP3 (*p* < 0.001).

In the non-diabetic subgroup, a similar pattern of improvement was observed, with the mean score decreasing from 62.50 pre-PRP to 47.92 post-PRP1, 33.33 post-PRP2, and 27.50 post-PRP3 (*p* < 0.001). Pairwise comparisons indicated significant improvements between pre-PRP and post-PRP1 (*p* = 0.002), post-PRP2 (*p* < 0.001), and post-PRP3 (*p* < 0.001). Furthermore, significant improvements in the scores were noted between the first and second PRPs (*p* = 0.001), as well as the first and third PRPs (*p* < 0.001). However, no significant difference was found between post-PRP2 and post-PRP3 scores (*p* = 0.978).

In the diabetic subgroup, the mean score decreased from 77.27 at pre-PRP to 53.03 post-PRP1, 45.45 post-PRP2, and 42.42 post-PRP3 (*p* < 0.001). Statistically significant improvements were observed between pre-treatment scores and all of the post-treatment scores (*p* = 0.048, *p* = 0.001, *p* < 0.001). However, no significant differences were found between the first and second PRPs (*p* = 1), respectively, the third PRP treatment (*p* = 0.645), or between the second and third PRP treatments (*p* = 1).

Inter-group analysis showed no significant differences between the diabetic and non-diabetic groups for pre-PRP and post-PRP1 mean scores (*p* = 0.401 and *p* = 0.44, respectively). Post-PRP2 and post-PRP3, non-diabetic patients reported significantly lower mean scores compared to their diabetic counterparts (*p* = 0.008 and *p* = 0.004, respectively).

(c) Question 3

Question 3 of the King’s Health Questionnaire (KHQ) assesses the extent to which urinary incontinence affects participants’ role limitations in daily activities.

In the overall study population, the mean score declined from 48.37 at baseline (pre-PRP) to 34.15 at post-PRP1, 26.31 at post-PRP2, and 19.28 at post-PRP3 (*p* < 0.0001). Among the non-diabetic subgroup, the mean score decreased from 47.29 (pre-PRP) to 32.92 (post-PRP1), 22.50 (post-PRP2), and 16.46 (post-PRP3) (*p* < 0.0001). Pairwise comparisons showed significant differences between pre-PRP and all post-treatment time points (*p* = 0.001 or lower), as well as between post-PRP1 and post-PRP2 (*p* = 0.021) and between post-PRP1 and post-PRP3 (*p* < 0.001). No statistically significant difference was observed between post-PRP2 and post-PRP3 (*p* = 0.445).

In the diabetic subgroup, mean scores decreased from 52.27 at baseline to 38.64 at post-PRP1, 40.15 at post-PRP2, and 29.55 at post-PRP3 (*p* = 0.01). Pairwise analysis showed no significant improvement between pre-PRP and post-PRP1 (*p* = 0.281) or between pre-PRP and post-PRP2 (*p* = 0.216). However, a considerable improvement was observed between pre-PRP and post-PRP3 (*p* = 0.002). No significant differences were found between post-PRP1, post-PRP2, and post-PRP3 scores (Supplementary Table S3).

No significant differences were observed between diabetic and non-diabetic groups at baseline (*p* = 0.586) or post-PRP1 (*p* = 0.367); however, post-PRP2 (*p* = 0.006) and post-PRP3 (*p* = 0.046), non-diabetic women reported significantly lower scores than diabetic women.

(d) Question 4

Question 4 of the King’s Health Questionnaire (KHQ) assesses participants’ physical limitations associated with urinary incontinence.

In the overall cohort, the mean score decreased from 34.31 at baseline (pre-PRP) to 23.86 at post-PRP1, 14.71 at post-PRP2, and 9.97 at post-PRP3 (*p* < 0.0001).

In the non-diabetic subgroup, scores declined from 34.38 (pre-PRP) to 24.17 (post-PRP1), 13.96 (post-PRP2), and 9.38 (post-PRP3) (*p* < 0.0001). Pairwise analysis revealed significant improvements between pre-PRP and all post-treatment time points (*p* ≤ 0.006), as well as between post-PRP1 and post-PRP2 (*p* = 0.004) and between post-PRP1 and post-PRP3 (*p* < 0.001). No significant difference was observed between post-PRP2 and post-PRP3 (*p* = 0.837).

In the diabetic subgroup, scores decreased from 34.09 at baseline to 22.73 at post-PRP1, 17.43 at post-PRP2, and 12.12 at post-PRP3 (*p* < 0.001). Pairwise comparisons showed significant improvements between pre-PRP and post-PRP2 (*p* = 0.014) and between pre-PRP and post-PRP3 (*p* = 0.001), whereas the difference between pre-PRP and post-PRP1 was not significant after adjustment (*p* = 0.189). Also, no significant differences were found between post-PRP1, post-PRP2, and post-PRP3 scores (Supplementary Table S4).

No statistically significant differences between diabetic and non-diabetic participants were observed at any time point (*p* = 0.89; *p* = 0.682; *p* = 0.795; *p* = 0.498, respectively).

(e) Question 5

Question 5 of the King’s Health Questionnaire (KHQ) evaluates participants’ social limitations related to urinary incontinence.

In the overall cohort, the mean score decreased from 33.33 at baseline (pre-PRP) to 22.22 at post-PRP1, 15.36 at post-PRP2, and 12.20 at post-PRP3 (*p* < 0.0001).

Among non-diabetic participants, scores decreased from 37.25 (pre-PRP) to 24.29 (post-PRP1), 16.99 (post-PRP2), and 13.83 (post-PRP3) (*p* < 0.001). Pairwise tests showed significant improvements versus baseline at all post-treatment time points (all *p* < 0.001), as well as between post-PRP1 and post-PRP2 (*p* = 0.001) and between post-PRP1 and post-PRP3 (*p* < 0.001); post-PRP2 vs. post-PRP3 was not significant (*p* = 0.572).

In the diabetic subgroup, scores decreased from 40.40 (baseline) to 25.76 (post-PRP1), 22.73 (post-PRP2), and 16.67 (post-PRP3) (*p* = 0.001). No significant changes were determined between pre-PRP and post-PRP1 (*p* = 0.22), but the scores significantly improved after the second (*p* = 0.01) and third PRP (*p* < 0.001); all comparisons among post-treatment time points were not significant (Supplementary Table S5).

No significant differences were observed between diabetics and non-diabetics at any time point; however, diabetics tended to have higher residual scores post-treatment.

(f) Question 6

Question 6 of the King’s Health Questionnaire (KHQ) addresses the impact of SUI on personal relationships. In the overall cohort, mean scores decreased modestly post-treatment from 73.04 at baseline to 69.44, 65.36, and 64.38 (*p* = 0.002).

Among non-diabetic participants, scores gradually improved from 66.87 pre-PRP to 63.54, 57.92, and 57.50 after treatment (*p* = 0.001). No significant change was observed after the first PRP treatment (*p* = 0.283), but after the second and third PRPs, the patients reported significant improvements (*p* = 0.006 and *p* = 0.001, respectively). Post-treatment comparisons were not significant (all *p* ≥ 0.283). In contrast, the diabetic subgroup showed no significant overall change across time (from 95.45 to 90.91, 92.42, and 89.39) (*p* = 0.91), and none of the pairwise comparisons reached significance after adjustment (all *p* = 1.00 or ≥ 0.86) (Supplementary Table S6).

Diabetic status was not associated with differences in the baseline scores (*p* = 0.064) or post-PRP1 (*p* = 0.084), but diabetic participants reported significantly higher scores post-PRP2 (*p* = 0.023) and post-PRP3 (*p* = 0.042).

(g) Question 7

Question 7 of the King’s Health Questionnaire (KHQ) assesses the emotional impact of SUI.

In the overall cohort, mean scores declined from 50.11 at baseline (pre-PRP) to 35.07 at post-PRP1, 24.73 at post-PRP2, and 19.06 at post-PRP3 (*p* < 0.001).

Among non-diabetic participants, scores decreased from 47.78 pre-PRP to 35.00 after the first treatment, 23.19 after the second treatment, and 17.64 after the third treatment (*p* < 0.001). Pairwise tests showed significant improvements versus baseline at all post-treatment time points (all *p* < 0.001), as well as between post-PRP1 and post-PRP2 (*p* < 0.001) and post-PRP1 and post-PRP3 (*p* < 0.001); post-PRP2 vs. post-PRP3 scores were not significantly different (*p* = 0.119).

In the diabetic subgroup, mean scores decreased from 58.59 at baseline to 35.35 at post-PRP1, 30.30 at post-PRP2, and 24.24 at post-PRP3 (*p* < 0.001), with significant improvements between the baseline and post-treatment scores (*p* < 0.01). The improvements between the first and third PRPs were also significant (*p* = 0.04), whereas no significant improvements were observed among the other time points (*p* = 0.72 and 0.88, respectively) (Supplementary Table S7).

Also, no significant differences between diabetic and non-diabetic participant reports were observed at any time point (*p* = 0.134; *p* = 0.945; *p* = 0.470; *p* = 0.467).

(h) Question 8

In the overall cohort, mean scores improved from 28.92 at baseline to 20.42 post-PRP1, 14.22 post-PRP2, and 10.46 post-PRP3 (*p* < 0.001).

In the non-diabetic subgroup, scores decreased from 24.37 to 17.29, 11.25, and 7.71 after the first, second, and third PRP treatments, respectively (*p* < 0.001). No significant improvements were observed after the first PRP treatment (*p* = 0.087), whereas improvements post-PRP2 and post-PRP3 versus baseline were significant (both *p* < 0.001). Significant improvements were also observed between the scores after the first and third PRP administrations (*p* = 0.006), while no significant changes were reported between the first and second or second and third PRPs (*p* = 0.161 and 1.000, respectively).

In the diabetic subgroup, scores declined from 45.45 (pre-PRP) to 31.82 (post-PRP1), 25.00 (post-PRP2), and 20.45 (post-PRP3) (*p* < 0.001), with significant improvements between baseline and the second (*p* = 0.01) and third PRPs (*p* < 0.001); comparisons among post-treatment time points were not significant (all *p* ≥ 0.41) (Supplementary Table S8).

Inter-group analysis indicated that diabetic participants experienced worse symptoms than non-diabetic participants at all time points (pre-PRP: *p* = 0.008; post-PRP1: 0.044; post-PRP2: 0.037; post-PRP3: 0.010).

(i) Question 9

In the overall cohort, mean scores decreased from 57.92 at baseline to 47.88 post-PRP1, 38.40 post-PRP2, and 33.58 post-PRP3 (*p* < 0.001), and all pairwise comparisons between scores at various timepoints were significant (*p* ≤ 0.047).

Among non-diabetic participants, scores declined from 55.94 to 46.35 post-PRP1, 37.40 post-PRP2, and 32.71 post-PRP3 (*p* < 0.001). Pairwise tests showed significant improvements versus baseline at all time points (all *p* ≤ 0.003), as well as between the first and second PRPs (*p* < 0.001) and post-PRP1 or the third PRP (*p* < 0.001); the difference between the scores post-PRP2 and post-PRP3 was not significant (*p* = 0.119).

In the diabetic subgroup, mean scores decreased from 65.15 at baseline to 53.41 post-PRP1, 42.05 post-PRP2, and 36.74 post-PRP3 (*p* < 0.001), with significant improvement compared to the baseline after the second and third PRPs (both *p* < 0.001), as well as between the first and third treatment (*p* = 0.001). All other score differences were not statistically significant (Supplementary Table S9).

Also, no significant differences were observed between diabetic and non-diabetic participants at any time point (*p* = 0.156; *p* = 0.374; *p* = 0.667; *p* = 0.742, respectively).

(j) Question 10

Question 10 of the King’s Health Questionnaire (KHQ) captures a symptom-specific severity score.

In the overall cohort, the severity of the symptoms’ perception decreased from 23.89 to 20.62 after the first, 18.36 after the second, and 16.85 after the third treatment (*p* < 0.001), and all pairwise comparisons were significant after Dunn–Bonferroni correction (all *p* < 0.001).

Among non-diabetic participants, means decreased from 23.51 (pre-PRP) to 20.46 (post-PRP1), 18.06 (post-PRP2), and 16.65 (post-PRP3) (*p* < 0.001), with significant improvements versus baseline at all time points (all *p* < 0.001), as well as between the first and second PRPs (*p* < 0.001), first and third PRPs (*p* < 0.001), or second and third PRPs (adj. *p* = 0.002).

In the diabetic subgroup, scores decreased from 25.27 to 21.18, 19.45, and 17.59 (*p* < 0.001), with significant improvements from baseline in each treatment (*p* ≤ 0.029), as well as between the first and third treatment (*p* < 0.001). No significant changes were captured between the first and second or second and third PRPs (*p* = 0.319 and 0.106, respectively) (Supplementary Table S10).

Also, no significant differences were reported between diabetics and non-diabetics at any time point (*p* = 0.071; 0.334; 0.148; and 0.251).

(k) Overall score

In the overall study population, the mean total score declined progressively from 436.50 (pre-PRP) to 354.06 (post-PRP1), 276.43 (post-PRP2), and 234.64 (post-PRP3).

**Figure 4:**
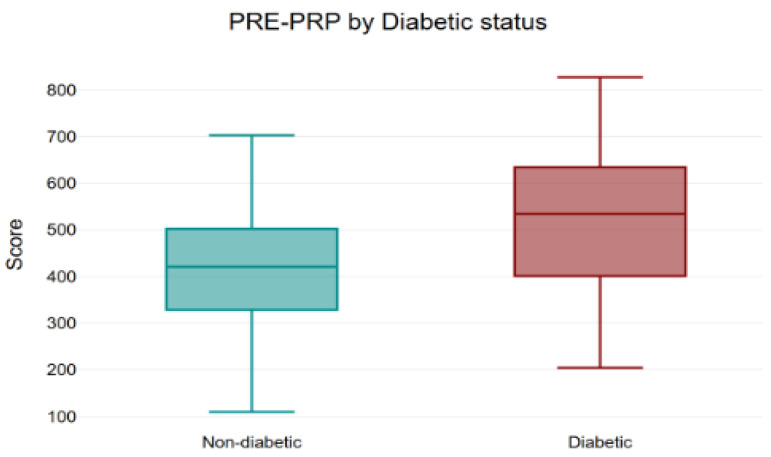
Mean baseline scores prior to PRP treatment, stratified by diabetic status. Boxplot showing the distribution of pre-PRP total questionnaire scores in diabetic and non-diabetic patients. Each box represents the interquartile range, with the median line indicating the central tendency and whiskers showing the minimum and maximum values. Diabetic patients presented higher baseline scores compared to non-diabetic patients, indicating more severe symptom burden before treatment (inter-group analysis *p* = 0.01). PRP: platelet-rich plasma.

**Figure 5:**
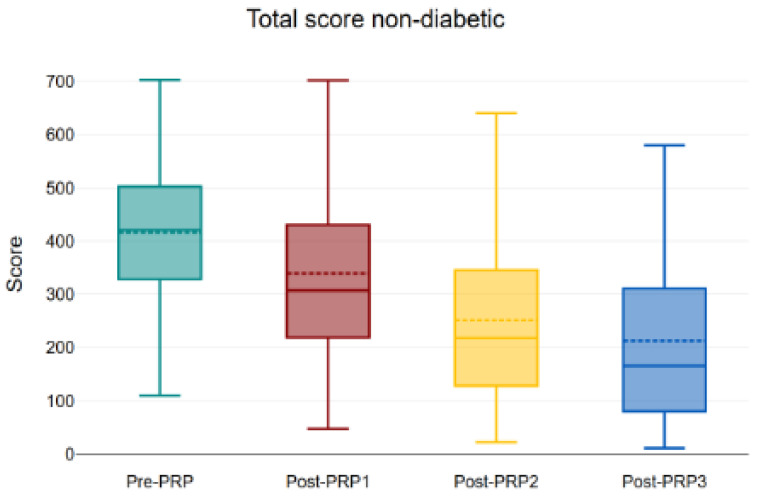
Evolution of total KHQ score in non-diabetic patients throughout the study (The box represents the interquartile range (IQR), the horizontal line indicates the median, and the dashed line represents the mean value. The whiskers show the minimum and maximum values within 1.5 × IQR). Boxplot illustrating the progression of total KHQ scores in non-diabetic patients before treatment (pre-PRP) and after each of the three PRP injections (post-PRP1, post-PRP2, and post-PRP3). A gradual reduction in median scores indicates improvement in symptoms and quality of life following sequential PRP treatments (inter-group analysis with all *p* < 0.001). PRP: platelet-rich plasma, KHQ: King’s Health Questionnaire.

Among non-diabetic participants, totals decreased from 416.12 to 339.21, 251.26, and 212.45, with a significant overall effect (*p* < 0.001) (Figure 4), as well as between baseline and each post-treatment visit (all *p* < 0.001) (Figure 5).

**Figure 6:**
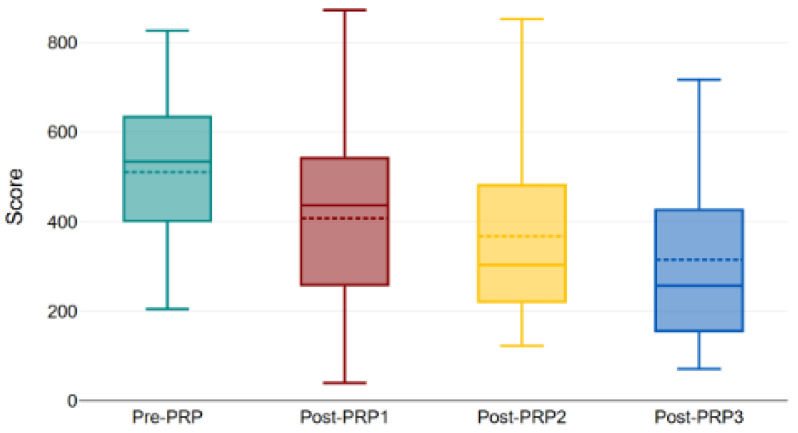
Evolution of total KHQ score in diabetic patients throughout the study (the box represents the interquartile range (IQR), the horizontal line indicates the median, and the dashed line shows the mean score. The whiskers represent the minimum and maximum values within 1.5 × IQR). Boxplot showing the variation in the total King’s Health Questionnaire (KHQ) scores in diabetic patients before treatment (pre-PRP) and after each PRP injection (post-PRP1, post-PRP2, and post-PRP3). The trend demonstrates progressive improvement in symptom severity and quality of life following sequential PRP treatments (*p* < 0.001). PRP: platelet-rich plasma.

**Figure 7:**
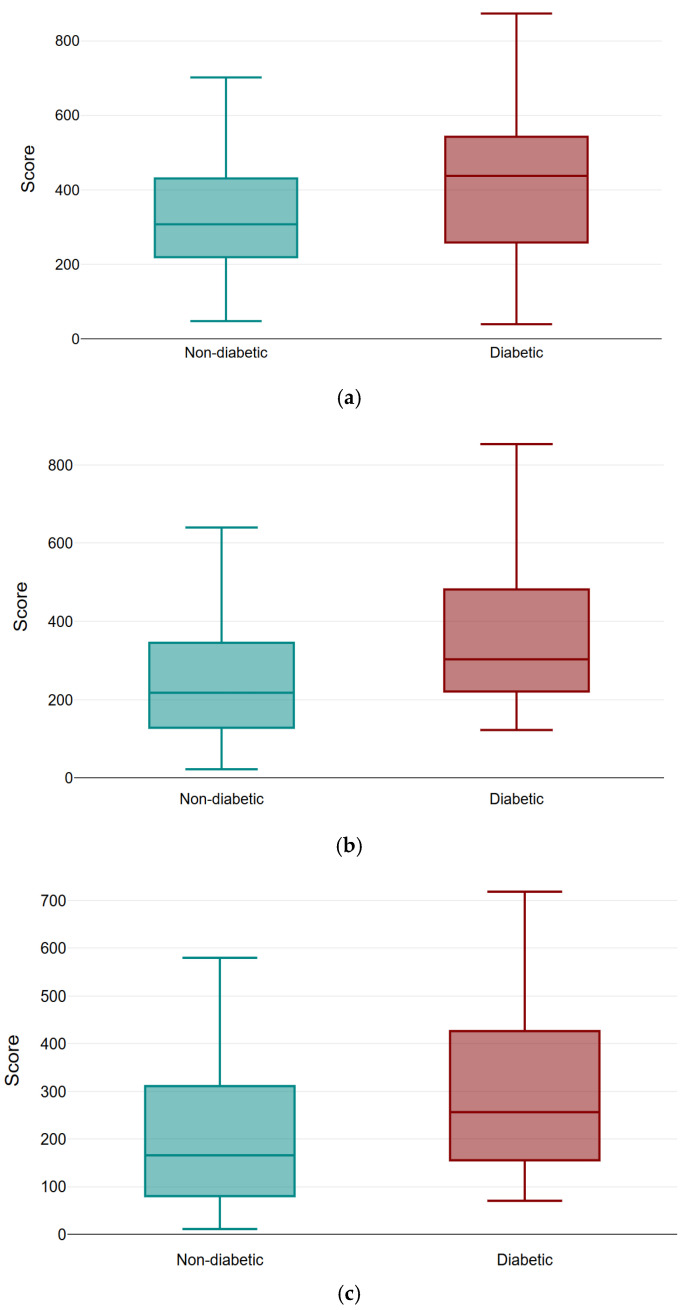
Comparison of mean KHQ scores after PRP sessions (1–3) in diabetic and non-diabetic patients. (**a**) Total score post PRP 1 by diabetic status, (**b**) Total score post PRP 2 by diabetic status, (**c**) Total score post PRP 3 by diabetic status. Boxplots illustrating the total KHQ scores in diabetic and non-diabetic patients following each PRP injection: (**a**) after PRP1, (**b**) after PRP2, and (**c**) after PRP3. Each box represents the interquartile range, with the horizontal line indicating the median and whiskers showing the minimum and maximum values. A consistent trend of greater improvement is observed in both groups across successive sessions, with non-diabetic patients showing slightly lower post-treatment scores at each time point. While no significant difference was found after PRP1 (*p* = 0.14), diabetic patients had significantly higher total scores at post-PRP2 (*p* = 0.01) and post-PRP3 (*p* = 0.02). PRP: platelet-rich plasma, KHQ: King’s Health Questionnaire.

In the diabetic subgroup, totals fell from 510.63 to 408.05, 367.94, and 315.32 with a significant overall change (*p* < 0.001) (Figure 4, Figure 6 and Figure 7).

Inter-group analysis indicated higher totals in diabetics at baseline (*p* = 0.01), no difference post-PRP1 (*p* = 0.14), and higher diabetic totals again at post-PRP2 (*p* = 0.01) and post-PRP3 (*p* = 0.02) (Figure 4 and Figure 7a–c).

In our cohort, pre- to post-treatment comparisons showed substantial gains in quality of life: the total KHQ score decreased by approximately 48% in non-diabetic women and 38% in women with diabetes, indicating benefit across both groups, though with a more modest response in diabetes (Table 4) (Appendix A).

### 3.2. Stamey Scale

The mean pre-treatment Stamey score was 1.49 ± 0.65, decreasing to 1.07 ± 0.35 after PRP therapy, with a mean improvement of 0.42 ± 0.55, indicating an overall reduction in SUI severity. Statistical comparison using the Wilcoxon signed-rank test demonstrated a highly significant improvement in Stamey scores for the entire cohort (*p* < 0.001).

Subgroup analyses revealed the following:

○ Among diabetic patients, PRP treatment resulted in a significant decrease in Stamey scores (*p* = 0.0027).
○ Among non-diabetic patients, a similarly significant reduction was observed (*p* < 0.001).

When comparing the magnitude of improvement (mean Stamey pre- vs. post-treatment) between diabetic and non-diabetic patients using the Mann–Whitney U test, no statistically significant difference was found (*p* = 0.96).

Among diabetic patients (n = 22), nine were insulin-dependent. In this subgroup, the mean Stamey score decreased from 1.33 ± 0.50 before treatment to 1 after PRP therapy, corresponding to a mean reduction of 0.33 ± 0.50; this change did not reach statistical significance (*p* = 0.083).

In contrast, diabetic patients not treated with insulin showed a significant improvement, with mean scores decreasing from 1.62 ± 0.65 to 1.15 ± 0.38 (*p* = 0.014).

A comparison of treatment response between insulin-dependent and non–insulin-dependent diabetic patients revealed no statistically significant difference in improvement (*p* = 0.58, Mann–Whitney U test) (Appendix B).

### 3.3. Safety Profile

No adverse events were reported in either the diabetic or non-diabetic groups throughout the study period. All PRP injections were well tolerated, with no cases of infection, bleeding, or prolonged local discomfort. This is particularly noteworthy for the diabetic cohort, given their increased baseline risk of impaired wound healing and susceptibility to infection.

## 4. Discussion

Despite the increasing prevalence of diabetes and SUI, little is known about the impact of diabetes on the efficacy of PRP in this patient subgroup [14]. This minority requires effective and accessible therapies, such as PRP treatment as a promising regenerative option for SUI, though its specific impact on diabetes patients remains largely unexplored [4,15].

Surgical management of SUI becomes a recommendation when conservative therapies (pelvic floor muscle training, pessaries, or bulking agents) prove insufficient. Among operative approaches, midurethral sling (MUS) procedures have emerged as the gold standard, favored for their high efficacy, minimal invasiveness, and relatively quick recovery; however, the use of synthetic mesh raises concerns about long-term safety and mesh-related complications. Surgeons and patients must weigh individual goals, anatomical considerations, and risk tolerance when selecting the most appropriate surgical intervention [16].

In our study, we evaluated a minimally invasive therapy with a modified protocol which addresses this underserved populations: women with diabetes and SUI. PRP treatment was associated with significant, progressive reductions in global KHQ scores, with a stepwise improvement in the reported scores after each subsequent PRP session [13]. However, while significant improvements were observed even after the first PRP treatment among non-diabetic patients in the majority of the scores, diabetic patients generally required more PRP administrations to achieve comparable outcomes. Non-diabetic women showed an early, graded improvement across most domains (general health and incontinence impact, role/physical limitations, social and emotional functioning, and sleep/energy) with most of the gain established by the second injection and little additional improvement added by the third PRP session. In women with diabetes, the perceived change was more gradual and uneven: several domains shifted only after the second or third session, between-treatment contrasts were smaller (notably for personal relationships), and energy-related burden tended to remain higher. Importantly, the symptom-focused measures (KHQ Q9 and Q10) improved in both groups, with no consistent inter-group gaps, suggesting that PRP reduces symptom severity irrespective of diabetes status, while translation into broader functional recovery occurs more slowly in diabetes.

Additionally, the persistence of between-group differences by the third session suggests that metabolic status may potentially moderate the pace or magnitude of QoL recovery, and that although diabetics benefit from these minimally invasive treatments, the outcomes are inferior to those achieved in the non-diabetic population. From a clinical perspective, this data supports the practice of patient counseling based on the fact that substantial improvement is expected by the second injection, with additional gains through the third, and counseling can motivate tailored expectations and potential protocol optimization for women with diabetes to narrow the residual gap.

The global score declined over time in both cohorts, but trajectories started from different baselines and diverged at later assessments: diabetics entered with a higher total burden and this remained higher by the second and third visits, whereas the groups were similar after the first injection. These patterns indicate that PRP confers meaningful, cumulative improvement in overall quality of life for all patients, yet women with diabetes improve at a slower pace, arguing for tailored counseling, attention to metabolic optimization, and supportive measures to narrow the residual difference by the end of the series.

There are some clinical implications this study can highlight: the most benefit is realized by the second injection for the average patient, with continued symptom improvement thereafter in severity-focused domains. These data support counseling non-diabetic women that early gains are expected and that a plateau is common beyond the second session. For women with diabetes, a slower or flatter trajectory in several functional domains should be anticipated. In our study, women with diabetes reported a greater baseline impact of SUI on QoL, as reflected by higher mean total KHQ scores compared with non-diabetic women (510.63 vs. 416.12) [13].

Regarding the Stamey score, our study found highly significant improvement in SUI severity for the entire cohort, as well as among the type 2 diabetic patients and non-diabetic patients. No statistically significant difference was found between diabetic and non-diabetic patients. This suggests that PRP therapy exerts a comparable therapeutic benefit in both groups, irrespective of diabetic status. Our study, although based on a small cohort, showed that the diabetics treated with insulin (9 patients) did not reach statistical significance in improving SUI, compared to the diabetics with no insulin treatment (13 patients).

SUI poses a substantial burden on both medical resources and societal economy. Risk factors for this type of pathology include menopause, childbirth, obesity, and constipation. These factors induce alterations in the anatomical components associated with urinary control, spanning the urethra, periurethral structures, and the pelvic nerve [7,8]. When injected periurethrally into the submucosal or surrounding connective tissue, PRP may promote the regeneration of the pubo-urethral ligaments, endopelvic fascia, and urethral sphincter complex, thereby improving urethral coaptation and resistance to intra-abdominal pressure [1,5,9,10].

The literature suggests that the presence of diabetes in women is frequently associated with impaired pelvic floor function, largely attributed to neuropathic damage and microvascular changes. Research on PRP therapy has highlighted its ability to enhance the structural integrity of pelvic floor tissues, suggesting a promising role in improving UI. Additionally, the regenerative properties of PRP may facilitate tissue repair and remodeling, potentially offering superior therapeutic benefits for diabetic patients compared to traditional treatment approaches [17,18].

One study reported links of diabetes with both UUI and MUI, another identified worsening glycemic control as a risk factor for SUI, and a third found associations with both UUI and SUI [14,19]. These relationships are thought to be mediated by diabetes’ microvascular damage and neuropathy, which can impair bladder innervation and alter detrusor muscle function [14]. In a cross-sectional population-based health study, UI prevalence was higher in women with diabetes (50.3%) compared to those without (39.3%); SUI was the most common subtype in both groups (20.3% vs. 18.1%). Women with diabetes also reported more frequent (mean score 2.4 vs. 1.8, *p* < 0.001) and more severe symptoms (mean total score 9.3 vs. 7.5, *p* < 0.001) than those without diabetes [14]. As our study revealed, women with diabetes had higher baseline scores—meaning a worse QoL than non-diabetic women.

Another connection between diabetes and SUI is represented by obesity, which increases intra-abdominal and pelvic pressure, often leading to SUI. The literature suggests that SUI treated surgically with midurethral slings has an increased risk of failure in women with diabetes and obesity. Weight reduction, particularly through dietary modification and physical exercise, has traditionally been endorsed as a fundamental component of the conservative therapeutic regimen for women experiencing SUI. Intriguingly, the literature states that lifestyle interventions, including exercise and dietary regulation, not only serve as preventive measures for the onset of diabetes, but also correlate with a consequential reduction in the overall prevalence of UI in women by nearly 50% [17,20,21].

A recent meta-analysis with randomized controlled trials assessed the effectiveness of PRP injections as a therapeutic modality for diabetic foot ulcers. The reviewed literature highlights a remarkable 100% healing rate with PRP therapy, exceeding the outcomes observed with conventional treatment modalities. Consequently, there is strong support for integrating PRP as a key element in the comprehensive management of diabetic foot ulcers [15].

Other uses of PRP treatment in diabetic patients suggest that the administration of intramyocardial PRP injection led to improvements in cardiac function and reperfusion, a reduction in infarct size, and an increase in ventricular wall thickness within a diabetic rat model mimicking acute myocardial infarction [22].

Protocols for PRP in women with SUI vary mainly by the number and timing of sessions, injection technique, and PRP preparation. Regarding preparation, most SUI studies used commercial kits, which are designed to produce leukocyte-reduced PRP, yet trials rarely report platelet dose, leukocyte differential, anticoagulant, or activation method in a standardized way. This heterogeneity may contribute to mixed efficacy signals across RCTs (one double-blind periurethral RCT reported symptom improvements over sham at 3–6 months, whereas a single-injection RCT found no difference vs. saline), underscoring the need to specify formulation and dosing per PRP reporting frameworks [1,23].

Commercial PRP kits were not employed in this study due to their high cost, which limits accessibility for many patients. By relying on a more economical preparation method, we sought to establish a protocol that could be realistically adopted in regions where financial constraints are a major concern. Our findings suggest that this approach is both effective and feasible, demonstrating successful outcomes not only in the general cohort but also in women with diabetes.

Published regimens range from single injections reported by Amirzargar et al. (2016) [24] and Tahoon et al. (2022) [25] to short-course series [10]. Apolikhina et al. (2018) [26] gave two injections of PRP-HA (hyaluronic acid) in 6/9 months, and most patients had resolution of stress leakage within 1–3 months [10]. Other schedules include two injections 4–6 weeks apart as described by Athanasiou et al. (2021) [27], and four monthly injections over 3 months reported by Chiang et al. (2022) [9] and Long et al. (2021) [28], while Daneshpajooh (2021) [29] reported one to three monthly injections over 2 months. Most of these studies used commercial kits [10].

The extended-interval design of this study contrasts with the more intensive short-term regimens in the literature and reflects a pragmatic approach intended to optimize patient compliance while still promoting connective tissue regeneration. Extended intervals between PRP injections were selected to minimize procedural burden and enhance tolerability in our cohort (mean age 58.63 years). In this age group, maintaining adherence to closely spaced treatments is often difficult, and longer spacing provided a practical compromise while still yielding measurable improvement.

The absence of hyaluronic acid in our protocol allows for direct evaluation of PRP’s regenerative potential in both diabetic and non-diabetic women, an important consideration given the impaired wound healing capacity traditionally associated with diabetes.

A large-scale study involving 4208 women diagnosed with SUI concluded that a higher frequency of SUI episodes was significantly associated with greater impairment in QoL, including both physical and mental domains. Moreover, they identified a possible correlation between SUI and increased levels of anxiety and depression, as well as work difficulties. Despite its debilitating impact, approximately 40% of women diagnosed with SUI do not seek medical intervention, highlighting the impact of this disease on women [3]. In our study, non-diabetics improved by 49%, diabetics by 38%, and the overall cohort by 46% on the KHQ total score across the three-injection series (see Table 4) [13].

Across all treatment sessions and scheduled follow-ups, no adverse events attributable to PRP were observed, including in the diabetic subgroup. There were no cases of infection, hematoma or clinically meaningful bleeding, urinary retention, urinary tract infection, hypersensitivity reactions, or pain persisting beyond the immediate post-procedure period, and no participant required unplanned care. Although the study was not powered to detect very rare events, these findings support a favorable safety profile of PRP for SUI in both diabetic and non-diabetic women.

### 4.1. Biological Rationale for Differential PRP Response in Diabetes

The response observed between diabetic and non-diabetic patients in our study may be explained by the pathophysiological alterations associated with diabetes. Chronic hyperglycemia is known to impair platelet activity, reduce growth factor bioavailability, and compromise angiogenesis and collagen synthesis. In addition, microvascular damage and persistent low-grade inflammation can limit local tissue regeneration and neuromuscular recovery. These mechanisms may collectively reduce the biological effectiveness of PRP in diabetic patients compared with non-diabetic individuals [17,18].

The mechanisms underlying the different responses to PRP therapy between diabetic and non-diabetic women are likely multifactorial. Diabetes mellitus is known to alter platelet function and vascular biology, even when platelet counts remain within normal limits. Chronic hyperglycemia promotes oxidative stress and non-enzymatic glycation of platelet membrane proteins, leading to abnormal activation patterns and reduced secretion of growth factors. These alterations may impair the angiogenic and regenerative potential of PRP, resulting in a weaker or delayed clinical response [30,31].

Endothelial dysfunction, a hallmark of diabetes, further contributes to this reduced efficacy. Hyperglycemia disrupts nitric oxide signaling and induces inflammatory and fibrotic changes within the microvasculature, limiting tissue perfusion and repair capacity. Experimental data also suggest that activation of the protein kinase C pathway in diabetic tissues can cause downstream resistance to PDGF (platelet-derived growth factor) signaling, which may blunt the regenerative cascade initiated by PRP [32,33,34,35].

In addition, diabetes is associated with chronic low-grade inflammation and extracellular matrix remodeling, both of which can compromise neuromuscular and connective tissue recovery in the urethral sphincter complex [36,37]. Studies in diabetic wound and neuropathy models have shown that PRP can still enhance healing, particularly when metabolic control is adequate, but its effectiveness diminishes with poor glycemic control or long-standing disease [38]. Clinical reports also suggest that variability in PRP effects may stem from differences in growth factor concentrations and patient metabolic status [39]. These findings support a temporal hypothesis: PRP may achieve the greatest benefit when applied earlier, before irreversible vascular, neural, or extracellular damage is established.

Taken together, these observations support the hypothesis that PRP therapy is most beneficial when administered early in the diabetic disease course, before irreversible neurovascular or fibrotic changes occur. In our study, all participants demonstrated normal fasting glucose and HbA1c values, indicating adequate metabolic control at the time of treatment. However, detailed data regarding disease duration and potential neurovascular complications were not available. The absence of these parameters limits a deeper understanding of how chronic metabolic alterations or diabetic microangiopathy might have influenced the regenerative response to PRP. Future studies should therefore incorporate comprehensive metabolic profiling, stratification by disease chronicity, and assessment of diabetes-related neurovascular involvement to better delineate their impact on PRP efficacy in women with SUI.

Although platelet concentration has long been regarded as a key determinant of PRP efficacy, clinical outcomes remain highly variable even when standardized preparation protocols are used. Increasing evidence indicates that therapeutic performance depends not only on platelet count but also on the biological quality and functional status of platelets. Factors such as platelet aging, oxidative stress, and systemic inflammation (particularly in metabolic disorders like diabetes) can impair platelet activation, growth factor release, and regenerative capacity, despite normal or even elevated platelet numbers. Moreover, in diabetes, sustained hyperglycemia promotes compensatory thrombopoiesis, reflecting an adaptive response to the heightened platelet turnover characteristic of the disease. Consequently, two PRP samples with identical platelet concentrations may differ markedly in biological activity [40]. Given that all participants in our study had normal platelet counts and underwent homogeneous PRP processing, we believe that differences in treatment outcomes are more likely related to platelet functionality.

In physiological conditions, insulin exerts a mild antiplatelet effect by stimulating endothelial nitric oxide synthesis and suppressing platelet aggregation. However, in patients with diabetes, particularly those requiring insulin therapy, this inhibitory action is often blunted due to platelet insulin resistance and chronic exposure to hyperglycemia-induced oxidative stress. Recent evidence shows that insulin-treated diabetic individuals display higher platelet reactivity and reduced responsiveness to antiplatelet agents compared with those managed with non-insulin therapies, reflecting a hyperactive platelet phenotype despite pharmacologic control of glycemia [41]. This dysfunctional platelet phenotype may partially explain the attenuated regenerative response observed in diabetic individuals, even when PRP is prepared under standardized conditions.

PRP from diabetic donors shows altered total protein and growth factor release profiles compared to non-diabetic donors, suggesting that comorbidities may affect platelet functional output even when platelet counts are similar. In this study, PRP from diabetics detected higher VEGF content—making autologous PRP application a promising treatment for diabetic foot ulcer [42].

In our cohort, among diabetic participants (n = 22), nine were insulin-dependent. This subgroup did not reach statistical significance in the mean reduction in the Stamey score, whereas the diabetic patients not treated with insulin demonstrated a significant improvement (*p* = 0.014). Although the between-group difference was not statistically significant (*p* = 0.58, Mann–Whitney U test), this pattern may suggest an attenuated regenerative response in insulin-treated women, possibly related to altered platelet function and impaired vascular reactivity associated with long-standing diabetes and insulin resistance.

Beyond diabetic foot ulcers, PRP has been explored in several diabetes-related conditions. In diabetic peripheral neuropathy, perineural PRP injections improved pain, sensory symptoms, and nerve conduction versus comparators in clinical studies and are the focus of ongoing work summarizing PRP across peripheral neuropathies [43,44]. In musculoskeletal disease, PRP (single centrifuged for 14 min at 1800 rpm, using bio-kits and activation with ACD-A anticoagulant) yielded superior pain relief and functional gains for adhesive capsulitis in diabetic cohorts compared with institution-based physical therapy; this observational study concluded that PRP represents a well-tolerated treatment for the management of this pathology in diabetic patients [45]. In periodontal disease among patients with type 2 diabetes (HbA1c > 7), adjunctive use of injectable PRF (platelet-rich fibrin) with scaling and root planing demonstrated similar positive outcomes as saline with scaling and root planing, in diabetes mellitus subjects [46]. In urology/sexual medicine, rat models and early human evidence suggest intracavernosal PRP may improve erectile function in diabetics via neurovascular repair mechanisms [47,48]. In the field of urogynecology, randomized data indicate periurethral PRP can improve SUI outcomes overall, although diabetes-specific stratification has not yet been reported—underscoring a key evidence gap for this population [1].

### 4.2. Limitations of the Study and Future Directives

The main limitation of the present study lies in the limited characterization of diabetic status among participants. Diabetes mellitus was recorded without stratification by glycemic control parameters, disease duration, or the presence of neurovascular complications and partially by treatment regimen (insulin-dependent or not). Although all participants had type 2 diabetes, normal fasting glucose values, and HbA1c not exceeding 6.5% at baseline, the absence of detailed metabolic profiling restricts the interpretation of diabetes-related differences in PRP responsiveness.

Moreover, the platelet concentration in each PRP sample was not quantified, although a standardized and uniform preparation protocol was applied to minimize technical variability and all participants of the study had platelet counts within the normal range. The diabetic subgroup was relatively small, which limits statistical power for detecting subtle between-group effects. As one of the first studies to explore PRP therapy in women with diabetes and SUI, our primary objective was to evaluate quality-of-life improvements rather than to conduct stratification. Future studies with larger diabetic cohorts, detailed assessment of glycemic and neurovascular parameters, and quantitative PRP characterization are needed to confirm these preliminary findings and provide stronger statistical power for subgroup analyses.

The non-randomized design without a sham or active control group limits the ability to establish definitive causality, and residual confounding cannot be excluded. Additionally, the chosen injection protocol has longer intervals between sessions than in most published studies which may limit generalizability. Another limitation of this study is that follow-up ended after the third injection, so durability beyond this horizon and retreatment needs are not defined.

Future studies should aim to include larger and more diverse diabetic cohorts, stratify participants by glycemic control, and incorporate both subjective and objective outcome measures. Randomized controlled trials comparing different PRP preparation methods, injection volumes, and session intervals could help to define optimal protocols for women with and without diabetes. Multicenter collaborations would further enhance external validity and facilitate the integration of PRP into standardized treatment algorithms for SUI.

Diabetes is biologically heterogeneous (type, duration, glycemic control, and microvascular complications), which amplifies confounding and inflates sample-size needs for stratified analyses. Higher comorbidity burden in diabetes (obesity, hypertension, and neuropathy) also increases exclusion rates and follow-up complexity. As a result, many trials have excluded patients with diabetes to limit variability, leaving a persistent evidence gap.

## 5. Conclusions

In women with SUI, PRP was associated with meaningful gains across KHQ domains and Stamey score. Functional scores (general health/impact, role/physical, and social–emotional) improved rapidly, being the largest by the second injection, with smaller changes thereafter, while symptom severity continued to decline through the third session. Responses differed by metabolic status: non-diabetic participants showed a graded reduction (≈49% in total KHQ), whereas diabetic participants improved more gradually from a higher baseline (≈38%). At time-matched follow-ups, symptom severity was essentially comparable between groups, indicating similar symptom control despite slower functional convergence in diabetes. Regarding the Stamey scores, our analysis showed that diabetics and non-diabetics improve with no difference, whereas the diabetics treated with insulin did not reach statistical significance in improving.

## Figures and Tables

**Table 1:** Inclusion and exclusion criteria.

Inclusion Criteria	Exclusion Criteria
Stress urinary incontinence reported by the patient and confirmed by gynecological examination (all degrees of SUI: mild, moderate, and severe)	Anatomical defect (anterior and/or central compartment prolapse > stage 1)
Contraindications for surgical treatment	Platelet disorders (qualitative or quantitative)
Patient’s preference for a non-surgical treatment	Sepsis
	Neoplasia
	Chronic treatment with NSAIDs

NSAIDs: non-steroidal anti-inflammatory drugs.

**Table 2:** Objectives of this study.

Objectives	Description
Primary Objective	To compare treatment outcomes using KHQ between diabetic and non-diabetic women treated with PRP for SUI. To compare the treatment outcomes using the Stamey scale.
Secondary Objectives	To evaluate overall symptom improvement and assess the occurrence of adverse events. Mean change in KHQ score within each group. Observe the difference in Stamey score between diabetics treated with insulin and diabetics without insulin.

KHQ: King Health’s Questionnaire.

**Table 3:** Participants of the study: age and parity.

Variable	Mean	Standard Deviation	Minimum	Maximum
Age (years)	58.63	10.77	38	82
Number of births	1.89	0.94	0	4

**Table 4:** Overall changes in our study.

Group	Pre-PRP Mean	Post-PRP3 Mean	Absolute Change	Percent Change
Non-diabetic (n = 80)	416.12	212.45	−203.67	−48.9%
Diabetic (n = 22)	510.63	315.32	−195.31	−38.3%
Entire cohort (n = 102)	436.50	234.64	−201.87	−46.2%

PRP: platelet-rich plasma; n: number.

## Data Availability

The data presented in this study are available from the corresponding author upon reasonable request due to privacy and ethical restrictions (GDPR, institutional approval). The King’s Health Questionnaire (KHQ) is licensed and not shareable; access should be requested from Mapi Research Trust (ePROVIDE).

## Supplementary Materials

The following supporting information can be downloaded at https://www.mdpi.com/article/10.3390/bioengineering12111179/s1, Table S1. Question 1. Table S2. Question 2. Table S3. Question 3. Table S4. Question 4. Table S5. Question 5. Table S6. Question 6. Table S7. Question 7. Table S8. Question 8. Table S9. Question 9. Table S10. Question 10. Table S11. Overall score.

## Appendix A

**Table A1:** Table of KHQ mean scores.

KHQ Item	Non-D Pre	Non-D PRP1	Non-D PRP2	Non-D PRP3	D Pre	D PRP1	D PRP2	D PRP3
Q1 General health	45.00	30.31	20.31	15.94	47.73	35.23	32.95	26.14
Q2 Incontinence impact	62.50	47.92	33.33	27.50	77.27	53.03	45.45	42.42
Q3 Role limitations	47.29	32.92	22.50	16.46	52.27	38.64	40.15	29.55
Q4 Physical limitations	34.38	24.17	13.96	9.38	34.09	22.73	17.43	12.12
Q5 Social limitations	31.39	21.25	13.33	10.97	40.40	25.76	22.73	16.67
Q6 Personal relationships	66.87	63.54	57.92	57.50	95.45	90.91	92.42	89.39
Q7 Emotions	47.78	35.00	23.19	17.64	58.59	35.35	30.30	24.24
Q8 Sleep/energy	24.37	17.29	11.25	7.71	45.45	31.82	25.00	20.45
Q9 Symptom severity (domain)	55.94	46.35	37.40	32.71	65.15	53.41	42.05	36.74
Q10 Symptom severity scale	23.51	20.46	18.06	16.65	25.27	21.18	19.45	17.59
Total KHQ score	416.12	339.21	251.26	212.45	510.63	408.05	367.94	315.32

Q: question; Non-D: non-diabetics; D: diabetics; PRP: platelet-rich plasma; KHQ: King Health’s Questionnaire.

## Appendix B

**Table A2:** Stamey scale on diabetics using insulin and diabetics without insulin.

Diabetics	N	Stamey Pre (Mean ± SD)	Stamey Post (Mean ± SD)	Mean Change (Mean ± SD)	*p*
With insulin	9	1.33 ± 0.50	1	0.33 ± 0.50	0.083
No insulin	13	1.62 ± 0.65	1.15 ± 0.38	0.46 ± 0.52	0.014
Mann–Whitney U comparison	22	*p* = 0.58			

N: number; SD: standard deviation; pre: pre-treatment; post: post-treatment; *p*: *p* value.
