# Supplementary material for: Does Diabetes Matter? The Efficacy of PRP on the Quality of Life in Stress Urinary Incontinence
Source: Bioengineering (Basel). 2025 Oct 29;12(11):1179. doi: 10.3390/bioengineering12111179 (PMC12649523; doi:10.3390/bioengineering12111179)
Supplement: Supplementary file 1 [file bioengineering-12-01179-s001.zip › bioengineering-3907915-supplementary.pdf]

Supplementary Table S1. Question 1

| Dunn-Bonferroni test | Test statistics | Standard error | Std. test statistics | p | Adj. p |
|---|---|---|---|---|---|
| Pre-PRP - Post-PRP1 | 0.93 | 0.18 | 5.12 | <.001 | <.001 |
| Pre-PRP - Post-PRP2 | 1.51 | 0.18 | 8.35 | <.001 | <.001 |
| Pre-PRP - Post-PRP3 | 1.8 | 0.18 | 9.95 | <.001 | <.001 |
| Post-PRP1 - Post-PRP2 | 0.58 | 0.18 | 3.23 | 0. 001 | 0. 005 |
| Post-PRP1 - Post-PRP3 | 0.87 | 0.18 | 4.83 | <.001 | <.001 |
| Post-PRP2 - Post-PRP3 | 0.29 | 0.18 | 1.6 | 0 .11 | 0. 439 |

Supplementary Table S2. Question 2

| | Test statistics | Standard error | Std. test statistics | p | Adj. p |
|---|---|---|---|---|---|
| Pre-PRP - Post-PRP1 | 0.78 | 0.18 | 4.31 | <.001 | <.001 |
| Pre-PRP - Post-PRP2 | 1.46 | 0.18 | 8.05 | <.001 | <.001 |
| Pre-PRP - Post-PRP3 | 1.67 | 0.18 | 9.22 | <.001 | <.001 |
| Post-PRP1 - Post-PRP2 | 0.68 | 0.18 | 3.74 | <.001 | 0.001 |
| Post-PRP1 - Post-PRP3 | 0.89 | 0.18 | 4.91 | <.001 | <.001 |
| Post-PRP2 - Post-PRP3 | 0.21 | 0.18 | 1.17 | 0.24 | 0.974 |

Supplementary Table S3. Question 3

| | Test statistics | Standard error | Std. test statistics | p | Adj. p |
|---|---|---|---|---|---|
| Pre-PRP - Post-PRP1 | 0.75 | 0.18 | 4.15 | <.001 | <.001 |
| Pre-PRP - Post-PRP2 | 1.21 | 0.18 | 6.67 | <.001 | <.001 |
| Pre-PRP - Post-PRP3 | 1.59 | 0.18 | 8.81 | <.001 | <.001 |
| Post-PRP1 - Post-PRP2 | 0.46 | 0.18 | 2.52 | 0.012 | 0.047 |
| Post-PRP1 - Post-PRP3 | 0.84 | 0.18 | 4.66 | <.001 | <.001 |
| Post-PRP2 - Post-PRP3 | 0.39 | 0.18 | 2.14 | 0.032 | 0.129 |

Supplementary Table S4. Question 4

| | Test statistics | Standard error | Std. test statistics | p | Adj. p |
|---|---|---|---|---|---|
| Pre-PRP - Post-PRP1 | 0.67 | 0.18 | 3.71 | <0.001 | 0.001 |
| Pre-PRP - Post-PRP2 | 1.27 | 0.18 | 7.05 | <0.001 | <0.001 |
| Pre-PRP - Post-PRP3 | 1.54 | 0.18 | 8.54 | <0.001 | <0.001 |
| Post-PRP1 - Post-PRP2 | 0.6 | 0.18 | 3.34 | 0.001 | 0.003 |
| Post-PRP1 - Post-PRP3 | 0.87 | 0.18 | 4.83 | <0.001 | <0.001 |
| Post-PRP2 - Post-PRP3 | 0.27 | 0.18 | 1.49 | 0.136 | 0.543 |

Supplementary Table S5. Question 5

| | Test statistics | Standard error | Std. test statistics | p | Adj. p |
|---|---|---|---|---|---|
| Pre-PRP - Post-PRP1 | 0.75 | 0.18 | 4.18 | <0.001 | <0.001 |

| | | | | | |
|---|---|---|---|---|---|
| Pre-PRP - Post-PRP2 | 1.38 | 0.18 | 7.62 | <0.001 | <0.001 |
| Pre-PRP - Post-PRP3 | 1.63 | 0.18 | 9.03 | <0.001 | <0.001 |
| Post-PRP1 - Post-PRP2 | 0.62 | 0.18 | 3.44 | 0.001 | 0.002 |
| Post-PRP1 - Post-PRP3 | 0.88 | 0.18 | 4.85 | <0.001 | <0.001 |
| Post-PRP2 - Post-PRP3 | 0.25 | 0.18 | 1.41 | 0.159 | 0.634 |

Supplementary Table S6. Question 6

| | Test statistics | Standard error | Std. test statistics | p | Adj. p |
|---|---|---|---|---|---|
| Pre-PRP - Post-PRP1 | 0.33 | 0.18 | 1.84 | 0.065 | 0.261 |
| Pre-PRP - Post-PRP2 | 0.54 | 0.18 | 2.98 | 0.003 | 0.011 |
| Pre-PRP - Post-PRP3 | 0.64 | 0.18 | 3.53 | <0.001 | 0.002 |
| Post-PRP1 - Post-PRP2 | 0.21 | 0.18 | 1.14 | 0.255 | 1 |
| Post-PRP1 - Post-PRP3 | 0.3 | 0.18 | 1.68 | 0.093 | 0.371 |
| Post-PRP2 - Post-PRP3 | 0.1 | 0.18 | 0.54 | 0.588 | 1 |

Supplementary Table S7. Question 7

| | Test statistics | Standard error | Std. test statistics | p | Adj. p |
|---|---|---|---|---|---|
| Pre-PRP - Post-PRP1 | 0.9 | 0.18 | 4.99 | <0.001 | <0.001 |
| Pre-PRP - Post-PRP2 | 1.72 | 0.18 | 9.49 | <0.001 | <0.001 |
| Pre-PRP - Post-PRP3 | 2.17 | 0.18 | 11.99 | <0.001 | <0.001 |
| Post-PRP1 - Post-PRP2 | 0.81 | 0.18 | 4.5 | <0.001 | <0.001 |
| Post-PRP1 - Post-PRP3 | 1.26 | 0.18 | 7 | <0.001 | <0.001 |
| Post-PRP2 - Post-PRP3 | 0.45 | 0.18 | 2.49 | 0.013 | 0.05 |

Supplementary Table S8. Question 8

| | Test statistics | Standard error | Std. test statistics | p | Adj. p |
|---|---|---|---|---|---|
| Pre-PRP - Post-PRP1 | 0.54 | 0.18 | 2.98 | 0.003 | 0.011 |
| Pre-PRP - Post-PRP2 | 0.96 | 0.18 | 5.31 | <0.001 | <0.001 |
| Pre-PRP - Post-PRP3 | 1.19 | 0.18 | 6.56 | <0.001 | <0.001 |
| Post-PRP1 - Post-PRP2 | 0.42 | 0.18 | 2.33 | 0.02 | 0.079 |
| Post-PRP1 - Post-PRP3 | 0.65 | 0.18 | 3.58 | <0.001 | 0.001 |
| Post-PRP2 - Post-PRP3 | 0.23 | 0.18 | 1.25 | 0.212 | 0.849 |

Supplementary Table S9. Question 9

| | Test statistics | Standard error | Std. test statistics | p | Adj. p |
|---|---|---|---|---|---|
| Pre-PRP - Post-PRP1 | 0.71 | 0.18 | 3.93 | <0.001 | <0.001 |
| Pre-PRP - Post-PRP2 | 1.69 | 0.18 | 9.36 | <0.001 | <0.001 |
| Pre-PRP - Post-PRP3 | 2.15 | 0.18 | 11.88 | <0.001 | <0.001 |
| Post-PRP1 - Post-PRP2 | 0.98 | 0.18 | 5.42 | <0.001 | <0.001 |

| | | | | | |
|---|---|---|---|---|---|
| Post-PRP1 - Post-PRP3 | 1.44 | 0.18 | 7.95 | <0.001 | <0.001 |
| Post-PRP2 - Post-PRP3 | 0.46 | 0.18 | 2.52 | 0.012 | 0.047 |

Supplementary Table S10. Question 10

| | Test statistics | Standard error | Std. test statistics | p | Adj. p |
|---|---|---|---|---|---|
| Pre-PRP - Post-PRP1 | 0.95 | 0.18 | 5.26 | <0.001 | <0.001 |
| Pre-PRP - Post-PRP2 | 1.87 | 0.18 | 10.36 | <0.001 | <0.001 |
| Pre-PRP - Post-PRP3 | 2.63 | 0.18 | 14.53 | <0.001 | <0.001 |
| Post-PRP1 - Post-PRP2 | 0.92 | 0.18 | 5.1 | <0.001 | <0.001 |
| Post-PRP1 - Post-PRP3 | 1.68 | 0.18 | 9.27 | <0.001 | <0.001 |
| Post-PRP2 - Post-PRP3 | 0.75 | 0.18 | 4.18 | <0.001 | <0.001 |

Supplementary Table S11. Overall score

Non-diabetics

| | Test statistics | Standard error | Std. test statistics | p | Adj. p |
|---|---|---|---|---|---|
| Pre-PRP - Post-PRP1 | 0.81 | 0.2 | 3.95 | <0.001 | <0.001 |
| Pre-PRP - Post-PRP2 | 1.75 | 0.2 | 8.57 | <0.001 | <0.001 |
| Pre-PRP - Post-PRP3 | 2.69 | 0.2 | 13.2 | <0.001 | <0.001 |
| Post-PRP1 - Post-PRP2 | 0.94 | 0.2 | 4.62 | <0.001 | <0.001 |
| Post-PRP1 - Post-PRP3 | 1.89 | 0.2 | 9.25 | <0.001 | <0.001 |
| Post-PRP2 - Post-PRP3 | 0.94 | 0.2 | 4.62 | <0.001 | <0.001 |

Diabetics

| | Test statistics | Standard error | Std. test statistics | p | Adj. p |
|---|---|---|---|---|---|
| Pre-PRP - Post-PRP1 | 1.09 | 0.39 | 2.8 | 0.005 | 0.02 |
| Pre-PRP - Post-PRP2 | 1.52 | 0.39 | 3.91 | <0.001 | <0.001 |
| Pre-PRP - Post-PRP3 | 2.66 | 0.39 | 6.83 | <0.001 | <0.001 |
| Post-PRP1 - Post-PRP2 | 0.43 | 0.39 | 1.11 | 0.267 | 1 |
| Post-PRP1 - Post-PRP3 | 1.57 | 0.39 | 4.03 | <0.001 | <0.001 |
| Post-PRP2 - Post-PRP3 | 1.14 | 0.39 | 2.92 | 0.004 | 0.014 |
